# Assessment of preoperative health-related quality of life in patients undergoing thyroidectomy based on patient-reported outcomes

**DOI:** 10.3389/fpsyg.2024.1329175

**Published:** 2024-08-08

**Authors:** Lei Liu, Yuqing Xiang, Lujing Xiong, Chao Li, Wei Dai, Jinchuan Hu, Chunyan Shui, Yuqiu Zhou, Xu Wang, Linjie Ma, Nan Xu, Bintao Hu, Yongcong Cai

**Affiliations:** ^1^Department of Head and Neck Surgery, Sichuan Clinical Research Center for Cancer, Sichuan Cancer Hospital and Institute, Sichuan Cancer Center, Affiliated Cancer Hospital of University of Electronic Science and Technology of China, Chengdu, China; ^2^School of Medicine, University of Electronic Science and Technology of China, Chengdu, China; ^3^Graduate School, Chengdu Medical College, Chengdu, China; ^4^Department of Thoracic Surgery, Sichuan Clinical Research Center for Cancer, Sichuan Cancer Hospital and Institute, Sichuan Cancer Center, Affiliated Cancer Hospital of University of Electronic Science and Technology of China, Chengdu, China

**Keywords:** patient-reported outcomes, thyroid neoplasm, health-related quality of life, anxiety, factors

## Abstract

**Introduction:**

To evaluate the preoperative health-related quality of life (HRQoL) and influencing factors of HRQoL in patients undergoing thyroidectomy based on patient-reported outcomes.

**Materials and methods:**

Patients who were diagnosed and treated in Sichuan Cancer Hospital from February 2022 to December 2022 and were scheduled to undergo thyroidectomy were included. Each participant completed the basic information questionnaire and patient-reported outcome assessment scales before surgery. HRQoL was assessed using the European Organization for Research and Treatment of Cancer Quality of Life Questionnaire-C30 (EORTC QLQ-C30), the Thyroid Cancer-Specific Quality of Life (THYCA-QoL), and the Hamilton Anxiety Scale (HAMA). The Wilcoxon rank sum test or the Kruskal-Wallis test was used to analyze the association between patient characteristics and HRQoL, and the variables with statistical significance were included in multiple linear regression analysis.

**Results:**

450 patients were included in the study. According to the analysis of the THYCA-QoL scores, the psychological subscale was the most complained about. Anxiety was the most common symptom of the HAMA. Factors associated with worse general QoL on the EORTC QLQ-C30 included nondiagnostic/unsatisfactory fine-needle aspiration (FNA) result. Planned lateral neck dissection and nondiagnostic/unsatisfactory FNA result were influential factors for preoperative anxiety. Males and longer sleep duration were associated with better thyroid cancer-specific QoL, better general QoL, and less anxiety.

**Conclusion:**

The preoperative HRQoL of patients undergoing thyroidectomy was generally good. Females, insufficient sleep duration, planned lateral neck dissection, and nondiagnostic/unsatisfactory FNA result were associated with worse preoperative HRQoL.

## Introduction

Thyroid cancer is the most common malignancy of the endocrine system and the head and neck (Li et al., 2020). The incidence of thyroid cancer has been increasing in recent years, with rates of 3.1 per 100,000 men and 10.1 per 100,000 women (Huang et al., 2023). Thyroid cancer is mainly treated with surgery and, if indicated, with thyrotropic-stimulating hormone suppression therapy, radioactive iodine therapy, etc. (National Comprehensive Cancer Network [NCCN], 2024). After standard treatment, patients with thyroid cancer have a good prognosis, with the highest 5-year relative survival rate of all malignancies at 98% (Wang et al., 2023). Thyroid cancer mortality has remained relatively stable at less than 1 in 100,000 in most countries (Pizzato et al., 2022; Chen D. W. et al., 2023).

Although thyroid cancer has a good prognosis, survival and mortality should not be the sole focus of clinical management. With the continuous development of medicine, especially in cancer treatment, improving patients’ health-related quality of life (HRQoL) is also key to clinical treatment (Li et al., 2020). Treatment and treatment-related complications (such as hypoparathyroidism, hoarseness, dysphagia, scar, etc.) have been shown to reduce postoperative HRQoL in patients (Haugen et al., 2016; Wang et al., 2018; Goswami et al., 2019; Chan et al., 2021; National Health Commission of the People’s Republic of China, 2022; Chen C. et al., 2023). However, at present, studies mainly focus on postoperative HRQoL, and there are few studies on monitoring preoperative symptoms and psychological status of patients with thyroid cancer. For the above reasons, this study evaluated HRQoL and its influencing factors in patients before thyroidectomy and compared it with factors influencing postoperative HRQoL to see if there are differences. As for the factors influencing preoperative HRQoL, we analyzed which aspects can be targeted to better help patients in the perioperative period, to further improve the treatment outcomes and patient satisfaction, and to reduce the impact of these factors on patients’ postoperative HRQoL.

## Materials and methods

### Research design

This study followed the Declaration of Helsinki and was approved by the Ethics Committee of Sichuan Cancer Hospital (2200058627). The investigators explained the purpose of the study in detail to the patients involved and provided them with secure electronic links to complete the questionnaires, and informed consent was obtained from all patients participating in the study. A total of 450 patients scheduled for thyroidectomy who were diagnosed and treated at Sichuan Cancer Hospital from February 2022 to December 2022 were included. The inclusion criteria for this study were as follows: (1) age 18–75 years; (2) able to complete the HRQoL scale independently; (3) planned to undergo thyroidectomy; (4) the waiting time for surgery was within 1 month; (5) in combination with the 2024 version of the National Comprehensive Cancer Network guidelines and the 2015 American Thyroid Association Management Guidelines (Haugen et al., 2016; National Comprehensive Cancer Network [NCCN], 2024), one of the following surgical indications was met: 1) the tumor was > 4 cm in diameter with compressive or structural symptoms; 2) with high clinical and/or radiographic suspicion of malignancy, some patients refused ultrasound-guided fine-needle aspiration (FNA) due to increased psychological pressure and required immediate surgery; 3) with high clinical and/or radiographic suspicion of malignancy and thyroid FNA suggestive of (a) carcinoma, (b) suspicious for carcinoma, (c) atypia/follicular lesion of undetermined significance, (d) follicular neoplasm, and (e) nondiagnostic/unsatisfactory, and some of these patients refused repeat FNA or molecular diagnostics due to psychological stress and required immediate surgery. Exclusion criteria included (1) previous history of neck surgery; (2) distant metastasis; (3) diagnosed with anxiety or depression; (4) thyroid dysfunction.

### Data collection

Patients were required to complete the preoperative personal information and patient-reported outcome assessment scales, which were independently completed by patients and included date of birth, gender, marital status, education, employment status, average sleep duration per day, FNA result, and HRQoL scales. The surgical procedure to be performed was completed by the medical staff. Before the patient completed the HRQoL scales, the medical staff voluntarily informed the patient of the intended surgical procedure. All patients completed electronic questionnaires on smartphones or tablets using research electronic data capture (REDCap), which is an international free and secure web application (Nipp et al., 2019). Our hospital introduced the system at the end of 2017 and installed it on our hospital’s server.

### Subjective assessment of HRQoL

We used three scales to assess the HRQoL of patients undergoing thyroidectomy: the European Organization for Research and Treatment of Cancer Quality of Life Questionnaire-C30 (EORTC QLQ-C30), the Thyroid Cancer-Specific Quality of Life (THYCA-QoL), and the Hamilton Anxiety Scale (HAMA) (Hamilton, 1959; Aaronson et al., 1993; Husson et al., 2013a; Supplementary Text 1). These three scales are widely used in HRQoL research, and this study obtained permission from the authors to use the above scales.

### Statistical analysis

All analyses were performed using SPSS version 26.0 statistical software (SPSS, Inc., Chicago, IL). Categorical variables are presented as frequencies and percentages, and continuous variables are described using mean, standard deviation (SD), median, and interquartile range (IQR). Because the sample size of FNA results indicating benign lesions or indeterminate lesions was small, we combined the benign lesion, atypia of undetermined significance, follicular lesions of undetermined significance, follicular neoplasm, and suspicious for a follicular neoplasm into one group. The Wilcoxon rank sum test or the Kruskal-Wallis test was used to examine the effect of sociodemographic characteristics (age, gender, marital status, education, employment status), lifestyle (sleep duration per day) and clinical factors (FNA results, and the type of surgery) on the EORTC-QLQ-C30 summary score, the THYCA-QoL summary score, and the HAMA summary score. Variables with a *P* < 0.05 were then subjected to multiple linear regression analysis, and *P* < 0.05 indicated that the difference was statistically significant. A variance inflation factor (VIF) of < 5 is acceptable for the identification of multicollinearity (Chen C. et al., 2023).

## Results

### Patient characteristics

**FIGURE 1:**
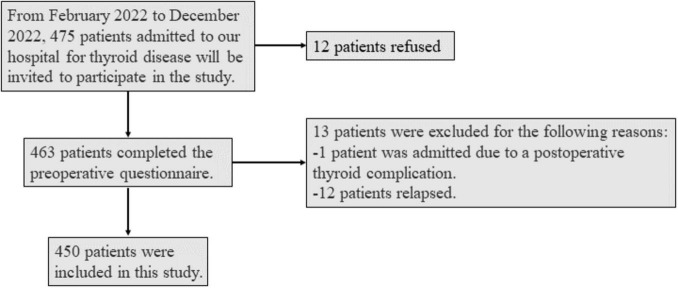
Flow-chart of the data collection process.

475 patients admitted for thyroid diseases were invited to the study, 12 patients refused, 13 patients were excluded due to re-admission for postoperative complications or recurrence, and the remaining valid data were 450 (Figure 1). The age range of the patients was 18.9–73.5 years, the median age was 40.9 years, and the female-to-male ratio was 3.4. As shown in Table 1, most of the patients were married (85.1%), had a stable job (76.2%), and slept > 6 hours (82.9%). Before surgery, the vast majority (92.0%) of patients accepted FNA. Regarding treatment options, 86.4% of patients planned to undergo open thyroidectomy, 32.2% of patients planned to undergo total thyroidectomy, and 14.9% of patients planned to undergo lateral neck dissection at the same time.

**TABLE 1:** General information and planned surgical methods of patients.

	*N*	%
**Gender**		
Female	347	77.1
Male	103	22.9
**Marital status**		
Married	383	85.1
Single/divorced/widowed	67	14.9
**Education**		
High school or below	168	37.3
Junior college or above	282	62.7
**Employment status**		
Employed	343	76.2
Unemployed	57	12.7
Retired	50	11.1
**Sleep duration (hours)**		
<5	24	5.3
5–6	53	11.8
6–7	170	37.8
>7	203	45.1
**FNA results**		
FNA wasn’t performed	36	8.0
Non-diagnostic/unsatisfactory	9	2.0
Benign	11	2.4
Atypia of undetermined significance/follicular lesions of undetermined significance	23	5.1
Follicular neoplasm/suspicious for a follicular neoplasm	9	2.0
Suspicious for malignancy	154	34.2
Malignant	208	46.2
Planned surgical approach		
Open thyroidectomy	389	86.4
Endoscopic thyroidectomy	61	13.6
Planned type of thyroidectomy		
Total thyroidectomy	145	32.2
Partial thyroidectomy	305	67.8
**Planned lateral neck dissection**		
None	383	85.1
Yes	67	14.9

FNA, fine-needle aspiration.

### Patient preoperative HRQoL

#### General HRQoL

The general HRQoL on the EORTC QLQ-C30 scores are shown in Table 2 and Supplementary Table 1. Among the five functional subscales, emotional functioning (69.6%) and cognitive functioning subscales (45.6%) were the two most common subscales with discomfort. The two functional subscales with the lowest scores were also emotional functioning (mean: 83.3, SD = 17.7) and cognitive functioning subscales (mean: 89.8, SD = 13.9). Of the nine symptom subscales, fatigue (46.7%) and insomnia (38.4%) were the most common. The two symptom subscales with the highest scores were also insomnia (mean: 15.0, SD = 20.8) and fatigue (mean: 11.0, SD = 14.9).

**TABLE 2:** Preoperative THYCA-QoL, EORTC QLQ-C30, and HAMA scores of patients.

Scale	Median (IQR)	Mean (SD)	Frequency (rating < 100) [*n* (%)]	Frequency (rating > 0) [*n* (%)]
**THYCA-QoL**				
Neuromuscular	0.0 (11.0)	8.0 (10.6)		212 (47.1)
Sympathetic	16.7 (16.7)	13.0 (14.8)		246 (54.7)
Throat/mouth problems	0.0 (11.0)	7.9 (10.2)		216 (48.0)
Psychological	8.3 (25.0)	14.9 (15.1)		311 (69.1)
Sensory	0.0 (16.7)	11.6 (14.5)		216 (48.0)
Felt chilly	0.0 (33.3)	19.0 (22.9)		215 (47.8)
Less interest in sex	33.3 (33.3)	19.6 (20.9)		232 (51.6)
**EORTC QLQ-C30**				
Global health status	83.3 (16.7)	74.9 (18.5)	382 (84.9)	
**Functional scale**				
Physical	100.0 (6.7)	95.7 (8.2)	144 (32.0)	
Social	100.0 (16.7)	92.5 (14.2)	127 (28.2)	
Cognitive	100.0 (16.7)	89.8 (13.9)	205 (45.6)	
Emotional	83.3 (25.0)	83.3 (17.7)	313 (69.6)	
**Symptom scale**				
Fatigue	0.0 (22.3)	11.0 (14.9)		210 (46.7)
Insomnia	0.0 (33.3)	15.0 (20.8)		173 (38.4)
Constipation	0.0 (0.0)	7.4 (15.1)		94 (20.9)
Financial difficulties	0.0 (0.0)	9.7 (18.5)		111 (24.7)
**HAMA**				
Anxious mood	1.0 (1.0)	0.8 (0.9)		263 (58.4)
Tension	1.0 (1.0)	0.7 (0.8)		232 (51.6)
Insomnia	1.0 (1.0)	0.7 (0.9)		232 (51.6)
Intellectual(cognitive)	0.0 (1.0)	0.6 (0.8)		215 (47.8)
Depressed mood	0.0 (1.0)	0.5 (0.8)		143 (31.8)

THYCA-QoL, Thyroid Cancer-Specific Quality of Life; EORTC QLQ-C30, Research and Treatment of Cancer Quality of Life Questionnaire-C30; HAMA, Hamilton Anxiety Scale.

As shown in Table 3, gender, sleep duration, and FNA results were significantly correlated (*P* < 0.05) with the EORTC QLQ-C30 summary score. Multiple linear regression analysis was performed on the above significant variables, which showed that females and nondiagnostic/unsatisfactory FNA results were the impact factors of the worse general HRQoL, whereas longer sleep duration was associated with better general HRQoL (*P* < 0.05; Table 4). These variables explained 10.8% of the variance in general HRQoL. The VIFs ranged from 1.020 to 3.653, indicating that multicollinearity was acceptable in this multivariable model.

**TABLE 3:** Bivariate analysis of patient characteristics with THYCA-QoL summary scores, EORTC QLQ-C30 summary scores, and HAMA summary scores.

Features	EORTC QLQ-C30 summary scores			THYCA-QoL summary scores			HAMA summary scores		
	Median (IQR)	Mean (SD)	*P*-value	Median (IQR)	Mean (SD)	*P*-value	Median (IQR)	Mean (SD)	*P*-value
Gender			**<0.001**			**<0.001**			**<0.001**
Female	93.5 (8.5)	92.1 (7.5)		17.6 (3.9)	18.2 (3.0)		5.0 (7.0)	6.3 (6.0)	
Male	96.6 (5.6)	95.2 (5.3)		15.8 (2.4)	16.2 (1.9)		2.0 (7.0)	4.2 (4.6)	
Age (years)			0.507			0.482			0.445
18–35	94.0 (7.7)	92.5 (7.4)		17.3 (4.3)	17.7 (3.0)		4.5 (7.0)	6.3 (6.0)	
36–64	94.7 (7.9)	93.0 (6.9)		17.0 (3.7)	17.6 (2.9)		4.0 (7.0)	5.6 (5.6)	
≥65	92.2 (10.9)	90.4 (10.8)		18.3 (4.5)	18.3 (3.2)		5.0 (10.0)	7.2 (8.4)	
Marital status			0.533			0.333			0.911
Married	94.4 (7.1)	93.0 (6.8)		17.2 (3.7)	17.6 (2.8)		4.0 (6.0)	5.7 (5.4)	
Single/ divorced/ widowed	93.6 (9.8)	91.6 (9.0)		17.3 (4.8)	18.3 (3.7)		4.0 (9.0)	6.7 (7.9)	
Education			0.626			0.463			0.929
High school or below	94.8 (8.3)	93.1 (6.5)		17.4 (3.7)	17.7 (2.8)		4.0 (8.0)	5.6 (5.2)	
Junior college or above	94.0 (7.5)	92.6 (7.6)		17.0 (4.0)	17.7 (3.0)		4.0 (6.0)	6.0 (6.2)	
Employment status			0.235			0.176			0.260
Employed	94.2 (7.6)	93.0 (6.7)		17.3 (3.8)	17.6 (2.9)		4.0 (6.0)	5.7 (5.7)	
Unemployed	95.6 (8.7)	92.9 (8.2)		17.1 (3.5)	17.6 (2.8)		4.0 (8.0)	5.6 (5.1)	
Retired	92.4 (8.3)	91.1 (8.6)		17.7 (3.7)	18.5 (3.4)		6.0 (7.0)	7.2 (6.9)	
Sleep duration (hours)			**<0.001**			**<0.001**			**0.002**
<5	89.8 (9.1)	89.5 (8.2)		18.5 (3.3)	18.8 (3.6)		8.5 (9.0)	8.4 (6.4)	
5–6	91.3 (10.1)	88.9 (9.8)		18.3 (3.5)	19.0 (3.1)		6.0 (9.0)	7.7 (6.8)	
6–7	93.9 (8.0)	92.8 (6.9)		16.9 (3.3)	17.6 (2.9)		4.0 (7.0)	6.0 (6.3)	
>7	95.5 (6.8)	94.1 (6.0)		16.8 (3.5)	17.3 (2.7)		4.0 (6.0)	5.9 (4.8)	
FNA results			**0.028**			0.160			**0.026**
FNA wasn’t performed	94.6 (7.8)	92.9 (7.9)		17.5 (3.9)	17.8 (3.3)		4.0 (7.0)	5.3 (6.0)	
Nondiagnostic/ unsatisfactory	87.8 (18.7)	81.8 (15.0)		19.8 (5.0)	20.4 (3.7)		9.0 (6.0)	11.2 (5.3)	
Combined group	95.5 (7.7)	93.3 (7.5)		17.3 (3.2)	17.3 (2.5)		3.0 (6.0)	5.3 (5.9)	
Suspicious for malignancy	94.1 (9.0)	92.9 (7.1)		17.0 (4.3)	17.7 (3.1)		5.0 (7.0)	5.9 (5.9)	
Malignant	94.2 (7.2)	93.0 (6.1)		17.2 (3.6)	17.6 (2.8)		4.0 (6.0)	5.8 (5.6)	
Planned surgical approach			0.380			0.959			0.597
Open thyroidectomy	94.7 (7.6)	92.9 (7.0)		17.3 (3.8)	17.7 (2.9)		4.0 (6.0)	5.8 (5.8)	
Endoscopic thyroidectomy	92.9 (8.2)	91.8 (8.1)		16.8 (3.5)	17.9 (3.3)		4.0 (8.0)	6.2 (5.9)	
Planned type of thyroidectomy			0.083			0.124			**0.027**
Total thyroidectomy	93.4 (7.6)	92.7 (5.6)		17.6 (3.5)	17.8 (2.6)		5.0 (7.0)	6.3 (5.2)	
Partial thyroidectomy	94.9 (8.5)	92.8 (7.8)		16.8 (4.0)	17.7 (3.1)		4.0 (7.0)	5.6 (6.1)	
Planned lateral neck dissection			0.064			0.850			**0.010**
No	94.7 (7.9)	92.9 (7.3)		17.2 (3.8)	17.7 (2.9)		2.0 (6.0)	5.6 (5.7)	
Yes	92.3 (9.3)	92.0 (6.1)		17.3 (3.7)	17.7 (2.9)		2.0 (9.0)	7.4 (6.1)	

FNA, fine-needle aspiration; THYCA-QoL, Thyroid Cancer-Specific Quality of Life; EORTC QLQ-C30, Research and Treatment of Cancer Quality of Life Questionnaire-C30; HAMA, Hamilton Anxiety Scale. Combined group: Benign/atypia of undetermined significance/follicular lesions of undetermined significance/follicular neoplasm/suspicious for a follicular neoplasm. Lower EORTC QLQ-C30 summary score, higher THYCA-QoL summary score, or higher HAMA summary score represents worse health-related quality of life. *P* < 0.05 is indicated in bold.

**TABLE 4:** Variables associated with THYCA-QoL summary scores, EORTC QLQ-C30 summary scores, and HAMA summary scores by multiple linear regression analysis.

Variable	EORTC QLQ-C30 summary scores			THYCA-QoL summary scores			HAMA summary scores		
	β -value	*t*-value	*P*-value	β -value	*t*-value	*P*-value	β -value	*t*-value	*P*-value
Gender (female as reference)	0.174	3.875	**<0.001**	−0.276	−6.155	**<0.001**	−0.156	−3.391	**0.001**
Sleep duration	0.198	4.401	**<0.001**	−0.158	−3.532	**<0.001**	−0.162	−3.509	**<0.001**
FNA results (not performed as reference)									
Nondiagnostic/unsatisfactory	−0.195	−3.938	**<0.001**	Not selected			0.124	2.450	**0.015**
Combined group	0.025	0.403	0.687	Not selected			−0.003	−0.045	0.964
Suspicious for malignancy	0.017	0.209	0.834	Not selected			0.025	0.294	0.769
Malignant	−0.014	−0.169	0.866	Not selected			0.046	0.520	0.603
Planned type of thyroidectomy (partial thyroidectomy as reference)	Not selected			Not selected			−0.009	−0.165	0.869
Planned lateral neck dissection (none as reference)	Not selected			Not selected			0.121	2.351	**0.019**
R2	0.120			0.106			0.083		
Adjusted R2	0.108			0.102			0.067		
VIF	1.020−3.653			1.004			1.022−3.804		

FNA, fine-needle aspiration; THYCA-QoL, Thyroid Cancer-Specific Quality of Life; EORTC QLQ-C30, Research and Treatment of Cancer Quality of Life Questionnaire-C30; HAMA, Hamilton Anxiety Scale; VIF, variance inflation factor. Combined group: Benign/atypia of undetermined significance/follicular lesions of undetermined significance/follicular neoplasm/suspicious for a follicular neoplasm. Lower EORTC QLQ-C30 summary score, higher THYCA-QoL summary score, or higher HAMA summary score represents worse health-related quality of life. *P* < 0.05 is indicated in bold.

#### Thyroid cancer-specific QoL

The thyroid cancer-specific QoL scores are shown in Table 2 and Supplementary Table 1. The preoperative thyroid cancer-specific QoL of the patients was generally good. Among all THYCA-QoL subscales, the two most common subscales were psychological problems (69.1%) and sympathetic problems (54.7%). The three subscales with the highest scores were less interest in sex (mean: 19.6, SD = 20.9), felt chilly (mean: 19.0, SD = 22.9), and psychological problems (mean: 14.9, SD = 15.1).

As shown in Table 3, gender and sleep duration were significantly correlated (*P* < 0.05) with the THYCA-QoL summary score. Multiple linear regression analysis of the above significant variables showed that females had worse thyroid cancer-specific QoL, while longer sleep duration was the influencing factor for better thyroid cancer-specific QoL (*P* < 0.05; Table 4). These variables explained 10.2% of the variance in thyroid cancer-specific HRQoL. The VIFs are both 1.004, indicating that multicollinearity was acceptable in this multivariable model.

#### Anxiety-related HRQoL

The anxiety-related HRQoL scores are shown in Table 2 and Supplementary Table 1. Among the patients’ self-reported HAMA scores, the three most common symptoms were anxious mood (58.4%), insomnia (51.6%), and tension (51.6%). The three subscales with the highest scores were anxious mood (mean: 0.8, SD = 0.9), insomnia (mean: 0.7, SD = 0.9), and tension (mean: 0.7, SD = 0.8).

As shown in Table 3, the results of the univariate analysis showed that gender, sleep duration, FNA results, planned type of thyroidectomy, and planned lateral neck dissection were significantly correlated with the HAMA summary score (*P* < 0.05). Statistically significant variables were then selected for subsequent multiple linear regression analysis, which showed that females, non-diagnostic/unsatisfactory FNA results, and planned lateral neck dissection were associated with more anxiety, while longer sleep duration was associated with less anxiety (*P* < 0.05; Table 4). These variables explained 6.7% of the variance in HAMA. The VIFs ranged from 1.022 to 3.804, indicating that multicollinearity was acceptable in this multivariable model.

## Discussion

This study investigated the preoperative HRQoL and HRQoL-related factors of patients undergoing thyroidectomy, but the preoperative HRQoL of these patients has not received much attention at home and abroad. However, it is necessary to understand patients’ preoperative symptoms, which not only helps to differentiate between pre-and post-treatment symptoms but also may improve treatment compliance and outcomes by controlling patients’ symptoms (Mendoza et al., 2019).

Our analysis of the EORTC QLQ-C30, the general HRQoL scale, showed that fatigue and insomnia were the most common preoperative symptom subscales. Previous studies have shown that these two symptoms are also the most common after thyroidectomy (Wang et al., 2018; Chan et al., 2021). This may be due to cancer-related fatigue or psychological distress. The study by Husson et al. found that psychological distress was associated with fatigue in thyroid cancer patients (Husson et al., 2013b).

We also did anxiety-related research on patients. The results of patients’ self-reported HAMA scores showed that anxiety was their most common preoperative symptom, which may be related to the patient’s fear of cancer diagnosis and treatment, fear of recurrence (Hedman et al., 2018; Song et al., 2021), or uncertainty about the side effects after treatment. A previous study found that patients with papillary thyroid cancer had higher levels of anxiety before surgery than at 3 months and 1 year after surgery (Song et al., 2021). Another study also found that anxiety levels were highest before initial treatment and decreased significantly thereafter (van Velsen et al., 2019). Clinicians should be aware that anxiety is an important concern for patients before surgery. Many patients, especially in underdeveloped areas, have no medical background, and the lack of knowledge about cancer leads to patients’ fear of cancer. After completing the anxiety scale, patients can be asked whether they need anxiety counseling, psychological help, or treatment counseling, and medical staff can provide cancer health education to patients in need, so that they can fully understand the tumor, reduce anxiety, and improve HRQoL.

Multiple linear regression analysis showed that gender was one of the factors influencing HRQoL: females were associated with lower EORTC QLQ-C30 summary scores, higher THYCA-QoL summary scores, and higher HAMA summary scores, suggesting that females have worse HRQoL than males. Similar results have been found in other studies of HRQoL after thyroid cancer surgery (Wang et al., 2018; Chen C. et al., 2023). Our analysis of the reasons may be as follows: (1) When faced with pressure, females are generally more likely to have stronger reactions and negative emotions such as anxiety or depression (Sze and Brunton, 2020); (2) The incision of open thyroidectomy is in a very prominent position, and females are more concerned about the neck scar (Campagnoli et al., 2023); (3) Some females who have not given birth may have increased concerns about fertility, and reports are suggesting that female with thyroid cancer have higher rates of infertility (Hirsch et al., 2023); (4) When a mother is diagnosed with cancer and planning surgery, she needs to focus on her illness, her family and her children, which can be very challenging for her (Kuswanto et al., 2018). Therefore, the above reasons may have strongly influenced females’ HRQoL. It is necessary for medical staff to monitor the psychological state of the patient before surgery and to inform the patient of the good prognosis of differentiated thyroid cancer. Secondly, with the continuous development of endoscopy and the Da Vinci robotic technology, these minimally invasive surgeries can perfectly balance aspects of radical tumor treatment, functional protection, and neck appearance (Prete et al., 2019; Kasemsiri et al., 2020), so patients can choose these surgical options to avoid neck scar problems. Patients who have open surgery can also choose a surgeon with better suturing techniques and use techniques (such as lasers, etc.) to reduce scarring (Chung et al., 2021), thereby reducing their psychological pressure about the neck appearance. In addition, studies have reported higher rates of infertility in patients with thyroid cancer, but clinicians can inform patients that they can have a normal pregnancy and childbirth even if they have cancer (Lee and Pearce, 2022).

In our research, lifestyle behaviors such as sleep also played an important role in patients’ preoperative HRQoL, with longer sleep duration being associated with better HRQoL. Another study also found that sufficient sleep time was associated with better HRQoL (Ge et al., 2019). Insufficient sleep can increase fatigue, depression, anxiety, and stress, as well as have adverse effects on the endocrine and metabolic systems (Ge et al., 2019; Scott et al., 2021). Patients with insufficient sleep can use sedative drugs and hypnotics to improve sleep, but these drugs can have side effects, drug tolerance, and dependence problems. Other methods include dietary therapy (such as dairy products, amino acids, or vitamin D), music-based intervention, acupressure, etc. (Chan and Lo, 2022; Tang et al., 2022; St-Onge et al., 2023). Improving patients’ sleep can help them cope better in the perioperative period and improve their HRQoL.

Our study analyzed the influence of whether preoperative FNA was performed and whether the FNA result is clear on patients’ HRQoL. Surprisingly, we found that patients with nondiagnostic/unsatisfactory FNA result had worse HRQoL. In this study, there were nine patients with nondiagnostic/unsatisfactory FNA result, and these patients were very anxious and had a strong desire for surgery. Possible reasons for this may be that these patients are still unclear about the pathological diagnosis of the disease after FNA, or they may find it difficult to choose a treatment plan, causing nervousness, anxiety, or other negative emotions. Clinicians should provide timely psychological counseling to the patient, explain to the patient in detail the possible reasons for the nondiagnostic/unsatisfactory FNA result, inform them of the next options and their advantages and disadvantages according to the radiographic and color ultrasound examinations, and suggest that they can undergo a repeat FNA or molecular diagnostics to confirm the diagnosis. Due to the limited number of patients with nondiagnostic/unsatisfactory FNA result in this study, the results may be biased, which needs to be verified by a larger sample size study in the future.

Regarding the choice of surgical approach, our study found that the planned endoscopic or open surgery and the planned total or partial thyroidectomy were not influential factors in patients’ preoperative HRQoL, possibly because these patients first focused on the diagnosis, radical treatment, recurrence, and metastasis of tumors, and then paid attention to the cervical scar cosmesis and surgical complications. However, studies of postoperative HRQoL have found that traditional open surgery and extended thyroidectomy are factors that influence patients’ poor HRQoL (Lee et al., 2016; Ryu et al., 2018; Nickel et al., 2019; Song et al., 2021), suggesting that surgery may have a negative impact on the patient’s postoperative HRQoL. Before surgery, clinicians can inform patients about surgical complications so that patients can fully anticipate the risks of surgery. Meanwhile, clinicians can provide patients with targeted preventive interventions after surgery to reduce the potential negative effects on patients’ HRQoL. We will continue to investigate these issues.

We found that lateral neck dissection was associated with preoperative anxiety. This may be because these patients are more concerned about the risk of tumor recurrence and distant metastasis, or they may be concerned that a larger surgical scope may involve longer incision, greater surgical risks, or more complications, which may lead to negative emotions such as anxiety. Surgeons can tell patients that the prognosis for differentiated thyroid cancer is good, with a distant metastasis rate of about 1%, and that even if patients have a recurrence or metastasis, they can survive for a long time after treatment (Lee et al., 2019; Nabhan et al., 2021; National Health Commission of the People’s Republic of China, 2022). At the same time, patients can be informed that long-term monitoring and follow-up will be performed after surgery to detect recurrent or metastatic tumors early, thereby reducing patient anxiety and improving HRQoL.

### Limitations

The study has the following limitations. First, the number of patients included was relatively small, and we did not recruit a normal population for comparison. Second, this study is a single-center analysis, which may not represent the overall preoperative HRQoL of patients undergoing thyroid surgery in China. Future larger multi-center studies are needed to address these limitations. Finally, this study only analyzed patients’ preoperative HRQoL, influencing factors, and possible treatments, and did not conduct any preoperative intervention. The next step is to explore the effect of specific interventions on HRQoL in patients undergoing thyroidectomy to improve patients’ HRQoL.

## Conclusion

Preoperative HRQoL was generally good for all patients undergoing thyroidectomy. However, females, shorter sleep duration, planned lateral neck dissection, and nondiagnostic/unsatisfactory FNA results were associated with worse preoperative HRQoL. Clinicians should provide more humane care and targeted interventions for these patients to reduce their preoperative anxiety, improve perioperative safety, and enhance their HRQoL. At the same time, medical staff should also communicate with patients about the progression of thyroid cancer or the low risk of recurrence of differentiated thyroid cancer, so that patients can understand the prognosis of thyroid cancer, reduce patients’ anxiety before surgery, and improve patients’ HRQoL. Our team’s following research will focus on the relationship between preoperative HRQoL and postoperative clinical outcomes in patients with thyroid cancer to provide evidence for early intervention in clinical symptoms.

## Data availability statement

The raw data supporting the conclusions of this article will be made available by the authors, without undue reservation.

## Ethics statement

The studies involving humans were approved by the Ethics Committee of Sichuan Cancer Hospital. The studies were conducted in accordance with the local legislation and institutional requirements. The participants provided their written informed consent to participate in this study.

## Author contributions

LL: Conceptualization, Formal analysis, Investigation, Methodology, Writing–original draft, Writing–review and editing. YX: Data curation, Formal analysis, Investigation, Methodology, Writing–review and editing. LX: Conceptualization, Data curation, Formal analysis, Methodology, Writing–review and editing. CL: Supervision, Writing–review and editing. WD: Methodology, Software, Writing–review and editing. JH: Data curation, Formal analysis, Investigation, Writing–review and editing. CS: Methodology, Project administration, Writing–review and editing. YZ: Data curation, Investigation, Writing–review and editing. XW: Methodology, Project administration, Writing–review and editing. LM: Investigation, Project administration, Writing–review and editing. NX: Formal analysis, Project administration, Writing–review and editing. BH: Investigation, Writing–review and editing. YC: Conceptualization, Funding acquisition, Supervision, Writing–review & editing.
