# Supplementary material for: Assessment of preoperative health-related quality of life in patients undergoing thyroidectomy based on patient-reported outcomes
Source: Front Psychol. 2024 Aug 8;15:1329175. doi: 10.3389/fpsyg.2024.1329175 (PMC11339645; doi:10.3389/fpsyg.2024.1329175)
Supplement: Supplementary file 1 [file Table_1.DOCX]

Supplementary Material

Supplementary Table 1. The more detailed preoperative THYCA-QoL, EORTC QLQ-C30, and HAMA scores of patients

| Scale | Median (IQR) | Mean (SD) | Frequency (rating<100) [n (%)] | Frequency (rating >0) [n (%)] |
| --- | --- | --- | --- | --- |
| THYCA-QoL |  |  |  |  |
| Neuromuscular | 0.0 (11.0) | 8.0 (10.6) |  | 212 (47.1) |
| Voice | 0.0 (0.0) | 4.5 (10.9) |  | 81 (18.0) |
| Concentration | 0.0 (16.7) | 7.8 (15.0) |  | 125 (27.8) |
| Sympathetic | 16.7 (16.7) | 13.0 (14.8) |  | 246 (54.7) |
| Throat/mouth problems | 0.0 (11.0) | 7.9 (10.2) |  | 216 (48.0) |
| Psychological | 8.3 (25.0) | 14.9 (15.1) |  | 311 (69.1) |
| Sensory | 0.0 (16.7) | 11.6 (14.5) |  | 216 (48.0) |
| Problems with scar | 0.0 (0.0) | 4.0 (13.9) |  | 42 (9.3) |
| Felt chilly | 0.0 (33.3) | 19.0 (22.9) |  | 215 (47.8) |
| Tingling hands/feet | 0.0 (0.0) | 3.4 (10.4) |  | 45 (10.0) |
| Gained weight | 0.0 (0.0) | 5.9 (14.9) |  | 69 (15.3) |
| Headache | 0.0 (33.3) | 9.6 (17.2) |  | 118 (26.2) |
| Less interest in sex | 33.3 (33.3) | 19.6 (20.9) |  | 232 (51.6) |
| EORTC QLQ-C30 |  |  |  |  |
| Global health status | 83.3 (16.7) | 74.9 (18.5) | 382 (84.9) |  |
| Functional scale |  |  |  |  |
| Role | 100.0 (0.0) | 98.6 (6.3) | 23 (5.1) |  |
| physical | 100.0 (6.7) | 95.7 (8.2) | 144 (32.0) |  |
| Social | 100.0 (16.7) | 92.5 (14.2) | 127 (28.2) |  |
| Cognitive | 100.0 (16.7) | 89.8 (13.9) | 205 (45.6) |  |
| Emotional | 83.3 (25.0) | 83.3 (17.7) | 313 (69.6) |  |
| Symptom scale |  |  |  |  |
| Fatigue | 0.0 (22.3) | 11.0 (14.9) |  | 210 (46.7) |
| Pain | 0.0 (0.0) | 3.1 (8.9) |  | 62 (13.8) |
| Nausea/vomiting | 0.0 (0.0) | 1.5 (6.4) |  | 29 (6.4) |
| Dyspnea | 0.0 (0.0) | 6.3 (15.3) |  | 74 (16.4) |
| Loss of appetite | 0.0 (0.0) | 5.2 (12.7) |  | 67 (14.9) |
| Insomnia | 0.0 (33.3) | 15.0 (20.8) |  | 173 (38.4) |
| Constipation | 0.0 (0.0) | 7.4 (15.1) |  | 94 (20.9) |
| Diarrhea | 0.0 (0.0) | 4.5 (11.8) |  | 59 (13.1) |
| Financial difficulties | 0.0 (0.0) | 9.7 (18.5) |  | 111 (24.7) |
| HAMA |  |  |  |  |
| Anxious mood | 1.0 (1.0) | 0.8 (0.9) |  | 263 (58.4) |
| Tension | 1.0 (1.0) | 0.7 (0.8) |  | 232 (51.6) |
| Fears | 0.0 (0.0) | 0.3 (0.7) |  | 102 (22.7) |
| Insomnia | 1.0 (1.0) | 0.7 (0.9) |  | 232 (51.6) |
| Intellectual (cognitive) | 0.0 (1.0) | 0.6 (0.8) |  | 215 (47.8) |
| Depressed mood | 0.0 (1.0) | 0.5 (0.8) |  | 143 (31.8) |
| General somatic(muscular) | 0.0 (0.0) | 0.3 (0.6) |  | 87 (19.3) |
| General somatic(sensory) | 0.0 (0.0) | 0.3 (0.5) |  | 108 (24.0) |
| Cardiovascular symptoms | 0.0 (0.0) | 0.2 (0.6) |  | 81 (18.0) |
| Respiratory symptoms | 0.0 (0.0) | 0.2 (0.6) |  | 85 (18.9) |
| Gastro-intestinal symptoms | 0.0 (1.0) | 0.3 (0.6) |  | 128 (28.4) |
| Genito-urinary symptoms | 0.0 (0.0) | 0.3 (0.6) |  | 95 (21.1) |
| Autonomic symptoms | 0.0 (1.0) | 0.4 (0.6) |  | 141 (31.3) |
| Behavior at interview | 0.0 (0.0) | 0.2 (0.5) |  | 70 (15.6) |

THYCA-QoL, Thyroid Cancer-Specific Quality of Life; EORTC QLQ-C30, Research and Treatment of Cancer Quality of Life Questionnaire-C30; HAMA, Hamilton Anxiety Scale.
