# Supplementary material for: Assessment of preoperative health-related quality of life in patients undergoing thyroidectomy based on patient-reported outcomes
Source: Front Psychol. 2024 Aug 8;15:1329175. doi: 10.3389/fpsyg.2024.1329175 (PMC11339645; doi:10.3389/fpsyg.2024.1329175)
Supplement: Supplementary file 2 [file Table_2.docx]

Supplementary Material

**Supplementary Text 1: Subjective assessment of health-related quality of life (HRQoL)**

The Thyroid Cancer-Specific Quality of Life (THYCA-QoL) is used to measure specific HRQoL in patients with thyroid cancer. The questionnaire consists of 24 items, with a time frame of the past week (except for the sexual interest item which is the past four weeks). THYCA-QoL includes seven symptom scales (neuromuscular, voice, concentration, sympathetic, throat/mouth, psychological and sensory problems) and six single items (problems with scars, felt chilly, tingling hands/feet, gained weight, headaches, and sexual interest). Each item is rated on a scale of not at all, a little, quite a bit, and very much, from 1 to 4, which is linearly converted to a standardized score ranging from 0 to 100 (Husson et al., 2013). A higher score on the sexual interest item indicates a higher interest in sexual behavior, and a higher score on the other items indicate more complaints about the symptom. Therefore, after the sexual interest item is scored in reverse, a higher score of this item indicates lower interest in sexual behavior (Chen et al., 2023). The Cronbach's alpha coefficients of the scale range from 0.45-0.82, and most of the THYCA-QoL scales are weakly correlated with the EORTC QLQ-C30 scales (r<0.40) (Husson et al., 2013).

The European Organization for Research and Treatment of Cancer Quality of Life Questionnaire-C30 (EORTC QLQ -C30) includes 30 questions that measure common questions about HRQoL in all cancer patients. It includes global health status (GHS); five functional scales: physical, role, cognitive, emotional, and social; three symptom scales: fatigue, pain, and nausea/vomiting; and 6 single items assessing common symptoms: dyspnea, loss of appetite, insomnia, constipation, diarrhea, and financial difficulty. The time frame for these questions is the most recent week. The answers to the first 28 questions have four levels: not at all, a little, quite a bit, and very much, on a scale of 1-4, respectively. The answers to the last two questions are divided into seven grades on a scale of 1-7. After a linear transformation, the range of scores for each domain is converted to a standardized score ranging from 0 to 100. A higher score on the functional scale and GHS means better function and general health, while a higher score on the symptom scale means more discomfort. The Cronbach's alpha coefficient of all scales was > 0.50, and there was a moderate correlation between scales, indicating that these scales assessed different parts of HRQoL (Aaronson et al., 1993). The summary score of the EORTC QLQ-C30 scale can be obtained by calculation, and its algorithm can be obtained through the website <http://groups.eortc.be/qol> (Giesinger et al., 2016).

The Hamilton Anxiety Scale (HAMA) was one of the first rating scales to measure the severity of anxiety symptoms. It consists of 14 symptom items, including psychological and physical symptoms: anxious mood; tension (including startle response, fatigability, restlessness); fears (including of the dark/strangers/crowds); insomnia; intellectual (poor memory/difficulty concentrating); depressed mood (including anhedonia); somatic symptoms (including aches and pains, stiffness, bruxism); sensory (including tinnitus, blurred vision); cardiovascular (including tachycardia and palpitations); respiratory (chest tightness, choking); gastrointestinal (including irritable bowel syndrome-type symptoms); genitourinary (including urinary frequency, loss of libido); autonomic (including dry mouth, tension headache) and observed behavior at interview (restless, fidgety, etc.). Scores range from 0 (not present) to 4 (severe) (Hamilton, 1959). The scale has sufficient reliability and validity (Maier et al., 1988).

**References:**

1. Aaronson, N. K., Ahmedzai, S., Bergman, B., Bullinger, M., Cull, A., Duez, N. J., et al. (1993). The European Organization for Research and Treatment of Cancer QLQ-C30: A Quality-of-Life Instrument for Use in International Clinical Trials in Oncology. *JNCI J. Natl. Cancer Inst.* 85, 365–376. doi: 10.1093/jnci/85.5.365
2. Chen, C., Cao, J., Wang, Y., Han, X., Zhang, Y., and Zhuang, S. (2023). Health-Related Quality of Life and Thyroid Cancer-Specific Symptoms in Patients Treated for Differentiated Thyroid Cancer: A Single-Center Cross-Sectional Survey from Mainland China. *Thyroid* 33, 474–483. doi: 10.1089/thy.2022.0490
3. Giesinger, J. M., Kieffer, J. M., Fayers, P. M., Groenvold, M., Petersen, M. Aa., Scott, N. W., et al. (2016). Replication and validation of higher order models demonstrated that a summary score for the EORTC QLQ-C30 is robust. *J. Clin. Epidemiol.* 69, 79–88. doi: 10.1016/j.jclinepi.2015.08.007
4. Hamilton, M. (1959). THE ASSESSMENT OF ANXIETY STATES BY RATING. *Br. J. Med. Psychol.* 32, 50–55. doi: 10.1111/j.2044-8341.1959.tb00467.x
5. Husson, O., Haak, H. R., Mols, F., Nieuwenhuijzen, G. A., Nieuwlaat, W.-A., Reemst, P. H., et al. (2013). Development of a disease-specific health-related quality of life questionnaire (THYCA-QoL) for thyroid cancer survivors. Acta Oncol. 52, 447–454. doi: 10.3109/0284186X.2012.718445
6. Maier, W., Buller, R., Philipp, M., and Heuser, I. (1988). The Hamilton Anxiety Scale: reliability, validity and sensitivity to change in anxiety and depressive disorders. Journal of affective disorders, 14(1), 61–68. doi:10.1016/0165-0327(88)90072-9
